# Targeting NPSR1-mediated Hippo-YAP1 dysregulation suppresses gastric cancer progression

**DOI:** 10.1007/s00018-026-06181-6

**Published:** 2026-04-09

**Authors:** Wenjing Qin, Mei Ma, Weidan Fang, Xian Wang, Bin Yu

**Affiliations:** 1 Department of General Surgery, The First Affiliated Hospital, Jiangxi Medical College, Nanchang University, Nanchang, 330006 PR China; 2 Department of Oncology, The First Affiliated Hospital, Jiangxi Medical College, Nanchang University, Nanchang, 330006 PR China

**Keywords:** Gastric cancer, NPSR1/ GPR154, YAP1, PP2A, cAMP/CREB

## Abstract

The targeting of G protein-coupled receptors (GPCRs) has emerged as a promising strategy for developing novel cancer therapies. Moreover, inactivation of the Hippo signaling pathway is recognized as a critical driver of gastric cancer (GC) progression. However, the precise molecular mechanisms underlying cross-talk between GPCRs and the Hippo pathway remain to be further investigated. Here, we identify NPSR1 as an oncogenic driver promoting GC advancement. NPSR1 is significantly overexpressed in GC tissues and negatively correlates with patient prognosis. Both in vivo and in vitro studies demonstrated that NPSR1 overexpression robustly accelerated GC cell proliferation and migration, while its silencing suppressed tumor progression. Mechanistically, our findings revealed a dual regulatory axis: (i) NPSR1 activated the cAMP/CREB pathway to enhance YAP1 transcription; and (ii) it directly facilitated phosphatase PP2Ac expression, thereby reducing YAP1 phosphorylation. This promotes the translocation of YAP1 from the cytoplasm to the nucleus, enhancing its oncogenic function. Our study uncovers a novel molecular link between NPSR1 hyperactivation and Hippo pathway suppression through coordinated transcriptional upregulation and post-translational modulation of YAP1. These results highlight NPSR1 as a potential therapeutic target for GC, offering a rationale for developing targeted inhibitors to restore Hippo signaling.

**Supplementary Information:**

The online version contains supplementary material available at 10.1007/s00018-026-06181-6.

## Introduction

Gastric cancer (GC) currently ranks as the fifth most prevalent malignancy worldwide and is the third leading cause of cancer-associated mortality, imposing a significant public health burden [1]. Epidemiological data indicate over one million new cases are diagnosed annually, with an alarming rise in GC incidence among younger populations under 50 years of age. It is important to note that the disease is frequently diagnosed at an advanced stage, in spite of recent advancements in early identification and cancer sensitivity screening, resulting in 768,000 deaths globally in 2020 [2]. The limited efficacy of current therapeutic approaches underscores an urgent need to elucidate the molecular mechanisms driving GC progression and to develop targeted intervention strategies.

G protein-coupled receptors (GPCRs), predominantly localized on the cell membrane, serve as pivotal mediators in signal transduction, orchestrating diverse cellular physiological and pathological processes. These receptors are structurally characterized by their distinctive seven transmembrane helical domains [3]. Upon ligand binding, GPCRs undergo conformational changes that facilitate the release of intracellularly bound heterotrimeric G proteins through exposure of their binding sites. The dissociated G protein subunits subsequently activate downstream effector molecules and initiate relevant signaling cascades, thereby modulating cellular functions. Emerging evidence from cancer research has demonstrated that GPCRs play crucial regulatory roles in cancer cell proliferation, survival, migration, and angiogenesis [4]. Despite significant therapeutic potential in targeted cancer therapy, the exploration of GPCRs as cancer targets remains relatively limited [5]. Currently, only a limited number of anticancer agents targeting GPCRs have been successfully translated into clinical practice [6]. Consequently, comprehensive investigations into the aberrant expression patterns of GPCRs and their underlying molecular mechanisms in cancer pathogenesis may unveil novel therapeutic targets, potentially revolutionizing the treatment landscape for cancer patients in the foreseeable future.

The Hippo signaling plays an important role in the physiological processes of the body, and its inactivation promotes cancer development and progression [7, 8], including the induction of cancer cell proliferation, metastasis, epithelial-mesenchymal transition (EMT) [9]. It is mainly mediated by a series of kinase cascade phosphorylation reactions that modify the transcriptional co-activators YAP1/TAZ [10, 11], thereby restricting their entry into the nucleus for transcriptional regulation of oncogene, such as CTGF, and CYR61 [12, 13]. Abnormalities in Hippo signaling can promote the development and metastasis of GC, such as the down-regulation of LATS1/2 expression in GC patients, suggesting an increased risk of metastasis and a decreased overall survival rate [14]. In addition, the aberrant increase of YAP1/TEADs in the nucleus was one of the important predictors of poor prognosis in GC [15, 16]. It has been showed that targeting the Hippo signaling pathway could bring potential therapeutic benefits to ERα + breast cancer patients (especially endocrine-resistant type) [17]. Therefore, in-depth investigation of the mechanism of Hippo pathway inactivation in GC is of great significance for its future precision treatment.

G protein-coupled receptor 154 (GPR154) belongs to the G protein-coupled receptor family, also known as NPSR1 (Neuropeptide S receptor1), for its first discovered ligand was neuropeptide S (NPS) [18]. NPSR1 has a variety of single-nucleotide polymorphisms (SNPs), which have been associated with diseases such as asthma, rheumatoid arthritis, and inflammatory bowel disease [19–21]. Current research on NPSR1 mainly focused on the nervous system, modulating behaviors including anxiety and cognitive learning [22]. However, there are few studies on NPSR1 in cancer, and reports have shown that NPSR1 might be a marker for neuroendocrine tumors [23], interfering with NPSR1 with compounds may affect the proliferation and migration of pancreatic cancer cells, and NPSR1 might be involved in the progression of thyroid cancer through the MAPK pathway [24]. Furthermore, the function and mechanism of NPSR1 in GC is still unclear. Here, we found that NPSR1 was abnormally highly expressed in GC tissues from both TCGA database and collected clinical samples, and its expression was significantly correlated with the prognosis. In addition, the proliferation, migration and invasion of GC cells were bidirectional regulation by NPSR1. Furthermore, we revealed NPSR1 inactivated Hippo pathway by regulating transcription and phosphorylation of YAP1. This study represents the first identification of NPSR1 as an oncogenic driver through Hippo/YAP1 axis deregulation in GC, positioning it as a dual therapeutic target for receptor inhibition and Hippo pathway reactivation strategies.

## Materials and methods

### Cell lines

Human gastric cancer cell lines MGC-803, MKN-45, HGC-27, and AGS-1 were cultured in RPMI-1640 medium containing 10% FBS (Fetal bovine serum, Gibco). All cells were cultured in a thermostat set at 37 °C and 5% CO2.

### Tissue microarrays and immunohistochemical staining (IHC)

GC tissue microarrays (HStmA180Su18) including samples from 94 cases paired 86 adjacent noncancerous tissue were obtained from Outdo Biotech Co., Ltd. (Shanghai, China). IHC followed the previously described protocol. In brief, the tissue was first deparaffinized, followed by antigen repair, then primary antibody incubation, further secondary antibody incubation and DAB color development, and finally hematoxylin re-staining. After successive dehydration, the slides were examined microscopically. In all cases, the intensity of the staining was evaluated three times independently of each other.

### RNA interference, plasmid construction, lentivirus, and transfectionRNA interf

A specific siRNA designed to knockdown NPSR1 as well as pcDNA3.1-NPSR1and pcDNA3.0-YAP1-EGFP plasmids were synthesized by GenePharma (Shanghai, China). siRNA sequences targeting NPSR1 was AAAGGAGTAGTAGAAGGAA [25], si-Control was TTCTCCGAACGTGTCACGT. PPP2CA siRNA sequence, sense (5’-3’): GGUGGCAAAUCACCAGAUATT, antisense (5’-3’): UAUCUGGUGAUUUGCCACCTT; Control siRNA sense (5’-3’):

UUCUCCGAACGUGUCACGUTT, antisense (5’-3’): ACGUGACACGUUCGGAGAATT. siRNA duplexes were transiently transfected into cells at a final concentration of 100 nM, using TurboFect transfection reagent (Thermo Scientific). Lentivirus used for NPSR1 and its control, namely Lv-NPSR1 and Lv-Control, were designed and synthesized from Genechem (Shanghai, China).

### RNA-seq and data analysis

High-throughput sequencing was conducted in accordance with established protocols. In brief, MKN-45 cells were treated with either NPSR1 or the control. Total RNA was then extracted using TRIzol Reagent (Invitrogen, USA) for RNA sequencing analysis, and a cDNA library was prepared for sequencing on the DNBSEQ platform at the Beijing Genomics Institute (Wuhan, China). Clean reads were aligned to the human reference genome (GRCh38.p13) using the HISAT2 (version 2.1.0) and Bowtie2 (version 2.4.2) software programs. Differential expression analysis was conducted and visualized through a volcano plot and hierarchical clustering heatmap. Additionally, KEGG pathway enrichment analysis was performed to identify significantly altered biological pathways. All bioinformatics analyses were systematically carried out using the Dr. Tom online bioinformatics platform (https://biosys.bgi.com/).

### CCK-8 and colony-formation assays

With regard to the CCK-8 assay, MGC-803, MKN-45, HGC-27, and AGS-1 cells in logarithmic growth phase were inoculated into 96-well plates at a density of 2 × 10^3^ cells per well. The cells were cultured in a cell culture incubator for 1 day, 2 days, 3 days, 4 days, and 5 days, respectively. Add 100 µl FBS-free medium including 10 µl of CCK8 reagent at the same time every day and continue to incubate for 2 h. Then, measure the absorbance value (OD) at 450 nm, and calculate the cells growth rate. To the colony-formation experiment, HGC-27, and AGS-1 cells were seeded with 700 cells in 6-well plates. After two weeks, colonies were fixed with methanol and stained by 0.5% crystal violet solution.

### Transwell assays and wound‑healing assay

To evaluate invasion and migration of GC cells, Transwell^®^ chamber (Corning, USA) coated with or without Matrigel (BD Science, USA) were applying in assays. MGC-803, MKN-45, HGC-27, and AGS-1 cells were used to seed into the upper chamber with 4 × 10^4^ cells with serum-free medium. Meanwhile, the bottom of the chamber encompassed 600 µl DMEM medium with 10% FBS. After 48 h, migrated or invaded cells into the bottom were fixed by methanol for 15 min and then stained with 1% crystal violet solution.

For wound‑healing assay, MGC-803, MKN-45, HGC-27, and AGS-1 cells were seeded in 6-well plates with 5 × 10^4^ cells/well. Cells forming monolayers after overnight, and wounds were scratched at the surface of the plates via sterile 10 µl pipette tips. Then the suspension cells were removed by phosphate-buffered saline (PBS) and rest of cells were cultured in serum-free DMEM medium in incubator. Images were captured by a phase-contrast microscope at 0 h and 24 h. the Image J software was used to calculate the wound closure percentage. Each independent assay was performed three times.

### RNA isolation and quantitative real-time PCR (RT-PCR)

Total RNA was isolated from cultured cells or xenograft tumor samples and extracted using TRIzol reagent (Invitrogen) according to the manufacturer’s instructions. After measurement, 1 µg of total RNA was synthesized into cDNA though PrimeScript™ RT reagent Kit with gDNA Erase (RR047A, Takara). The RT-PCR with SYBR Green PCR Master Mix (Takara) was executed on CFX96 Real-Time PCR Detection System (Bio-Rad). Relative gene expression was evaluated by 2^−ΔΔCt^ normalized to GAPDH. The primer sequences are listed in Supplementary Information, Table S1.

### In vivo xenograft growth assay

All animal care and handling procedures were carried out in accordance with the Guidelines for the Care and Use of Laboratory Animals of the First Affiliated Hospital of Nanchang University. Female BALB/c nude mice, 6–8 weeks of age, were purchased from Hunan SJA Laboratory Animal Co., Ltd (Hunan, China). Selecting 1 × 10^7^ Lv-NPSR1 or Lv-Control-treated MGC-803 cells were subcutaneously inoculated into the flanks of each nude mice. The sizes of tumors were measured every week by electronic digital calipers (Thermo Scientific), and the xenograft tumors were excised, photographed and weighed at 5 weeks.

### Protein extraction and immunoblotting

Briefly, after lysing the collected cells with RIPA buffer, the extracted total proteins were separated by 10% SDS-PAGE and transferred to PVDF (polyvinylidene difluoride) membranes. After blocking in PBST-5% non-fat milk, the membranes were incubated sequentially with primary and secondary antibodies. The visualization of the proteins was conducted with the ECL detection system (Bio-Rad). The antibodies information listed in Supplementary Information, Table S2.

### cAMP assay

The HGC-27 cells were cultured in 6-well plates and pcDNA3.1-NPSR1 or pcDNA3.1-Control were transfected into cells respectively after overnight. To assess intracellular cAMP amount, 48-hour transfected cells were harvested to extract total proteins. The cAMP levels were measured in accordance with the manufacturer’s protocol of cAMP Parameter Assay Kit (KGE002B, R&D Systems, Germany). Samples prepared and tested in triplicate.

### Statistical analysis

All the data were represented as the mean ± standard deviation. The Student’s t-test, one-way ANOVA, and two-way ANOVA were adopted for the statistical analyses. *P* < 0.05 was considered statistically significant. And * indicates *P* < 0.05, ** indicates *P* < 0.01, and *** indicates *P* < 0.001.

## Results

### NPSR1 level is upregulated in GC, and its high expression is a marker of poor prognosis

**Fig. 1:**
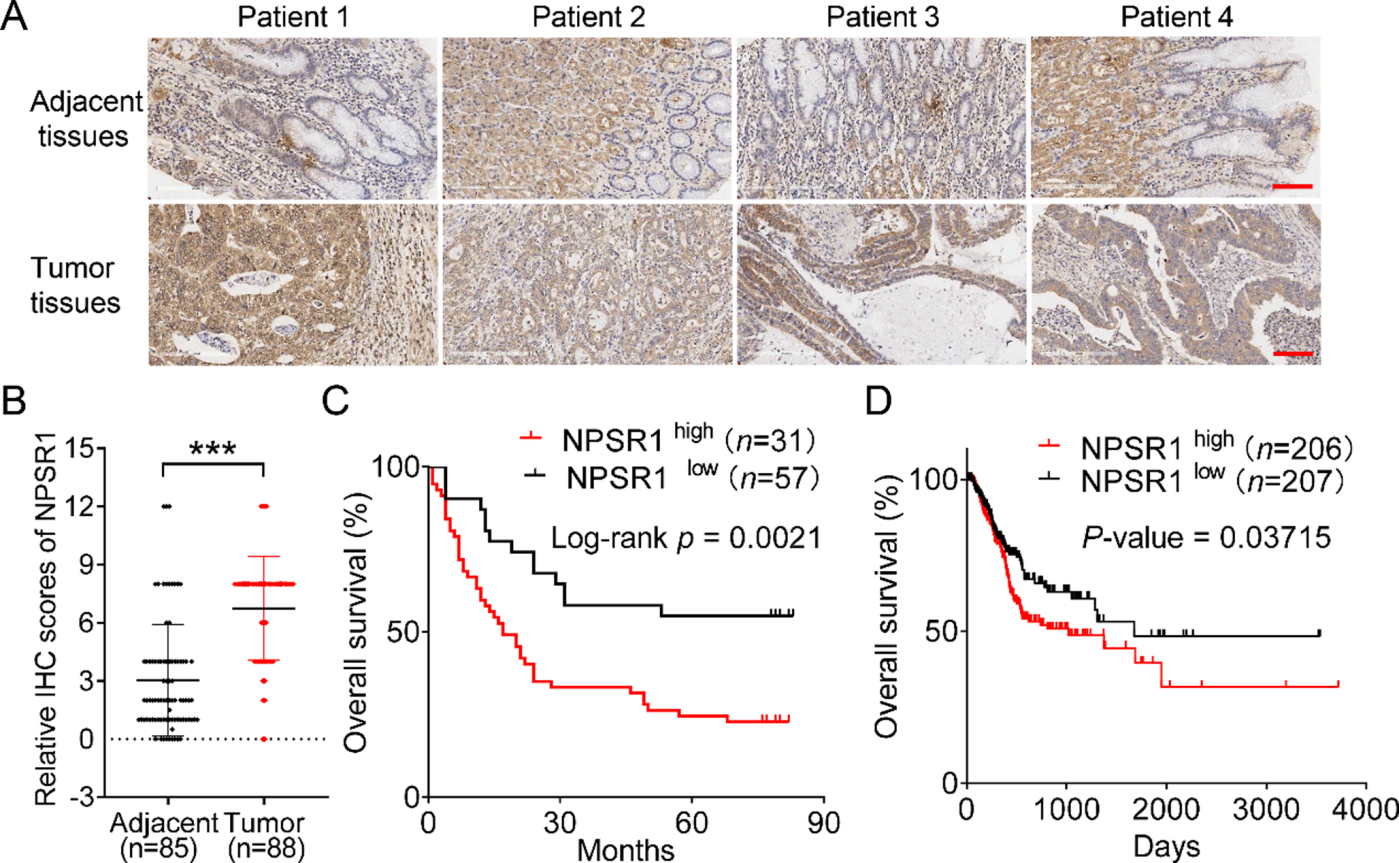
NPSR1 level is upregulated in GC, and its high expression is a marker of poor prognosis. **A**. Representative images of tissue microarrays of NPSR1 in GC (n = 94; upper panel) or adjacent noncancerous (n = 86; bottom panel) tissues. Scale bar: 100 µm. **B**. Immunohistochemical staining (IHC) score of NPSR1 in GC (n = 88) and adjacent noncancerous (n = 85) tissues, Student’s t test, ***P < 0.001. **C**. Overall survival (OS) of high NPSR1 (n = 31) and low NPSR1 (n = 57) GC patients was compared using Kaplan-Meier survival analysis, P = 0.0021. **D**. Analysis of the TCGA database showed the relationship between the prognosis of 413 patients and the expression of NPSR1, P = 0.03715.

To uncover therapeutic targets in GC, we interrogated the TCGA transcriptomic dataset with a focus on GPCRs, identifying NPSR1 as a candidate driver of GC progression. Our analysis revealed that NPSR1/GPR154 exhibited aberrant overexpression across diverse tumor tissues and was significantly upregulated in GC tissues compared to non-tumor controls (Figure S1A). Then, we examined in clinical samples from pathological tissue of GC to detect NPSR1 expression by tissue microarray and the results also showed that NPSR1 was significantly higher expression in cancer tissues comparative to adjacent normal parts (Fig. 1A-B; Table 1). In addition, patients in higher stages or metastasis had more NPSR1 expression compared with the normal group (Figure S1B-C, Table 2). To evaluate the clinical significance of NPSR1 upregulation, we performed Kaplan-Meier survival analysis stratified by immunohistochemistry-defined high/low expression thresholds. Patients in the high-expression cohort displayed significantly poorer overall survival (OS) compared to low-expression counterparts (Fig. 1C; Table 3). These findings were corroborated in TCGA data, where elevated NPSR1 mRNA levels correlated with reduced OS across independent GC cohorts (Fig. 1D). Furthermore, NPSR1 expression in GC cell lines was obviously higher than in normal GC cells (Figure S1D). The above results indicate that NPSR1 had a critical role in GC tumorigenesis and might represent a clinically actionable therapeutic target.

**Table 1:** Differential expression of NPSR1 in Gastric cancer and adjacent tissues

	NPSR1 expression		Chi-square Value	*p* value
	High(%)	Low(%)		
Gastric cancer	57	31	46.271	< 0.001
Adjacent tissues	12	73		

**Table 2:** Correlation between NPSR1 expression and clinicopathological characteristics

	Variables	NPSR1 expression		Total	χ2	*p* value
		Low	High			
Age (year)					0.704	0.402
	<=55	9	12	21		
	>55	22	45	67		
Sex					0.859	0.354
	Female	14	20	34		
	male	17	37	54		
Grade					1.931	0.165
	I-II	12	14	26		
	III-IV	19	43	62		
T stage					0.234	0.890
	I-II	5	9	14		
	III	17	34	51		
	IV	9	14	23		
N stage					8.675	0.034
	N0	10	12	22		
	N1	8	6	14		
	N2	6	9	15		
	N3	7	30	37		
M stage					0.949	0.330
	M0	31	53	84		
	M1	0	4	4		
TNM stage					4.967	0.174
	I	3	5	8		
	II	14	15	29		
	III	14	33	47		
	IV	0	4	4		

**Table 3:** Univariate and multivariate analyses of the factors correlated with overall survival of GC patients

Variables	Univariate analysis				Multivariate analysis			
	HR	95%CI	*p* value		HR	95%CI	*p* value	
Expression	2.473	1.351–4.525	0.003		1.556	0.806–3.002	0.188	
Sex	0.688	0.418–1.134	0.143					
Grade	2.049	1.088–3.860	0.026		1.229	0.626–2.415	0.549	
Age	0.837	0.483–1.450	0.526					
T stage	2.049	1.373–3.058	< 0.001		1.333	0.799–2.226	0.271	
N stage	2.49	1.873–3.326	< 0.001		2.122	1.215–3.705	0.008	
M stage	3.911	1.372–11.144	0.011		1.371	0.466–4.030	0.566	
TNM stage	5.493	2.978–10.131	< 0.001		1.260	0.399–3.979	0.693	

* Statistically significant (*p* < 0.05)

### NPSR1 is critical for the cell proliferation and migration of GC

**Fig. 2:**
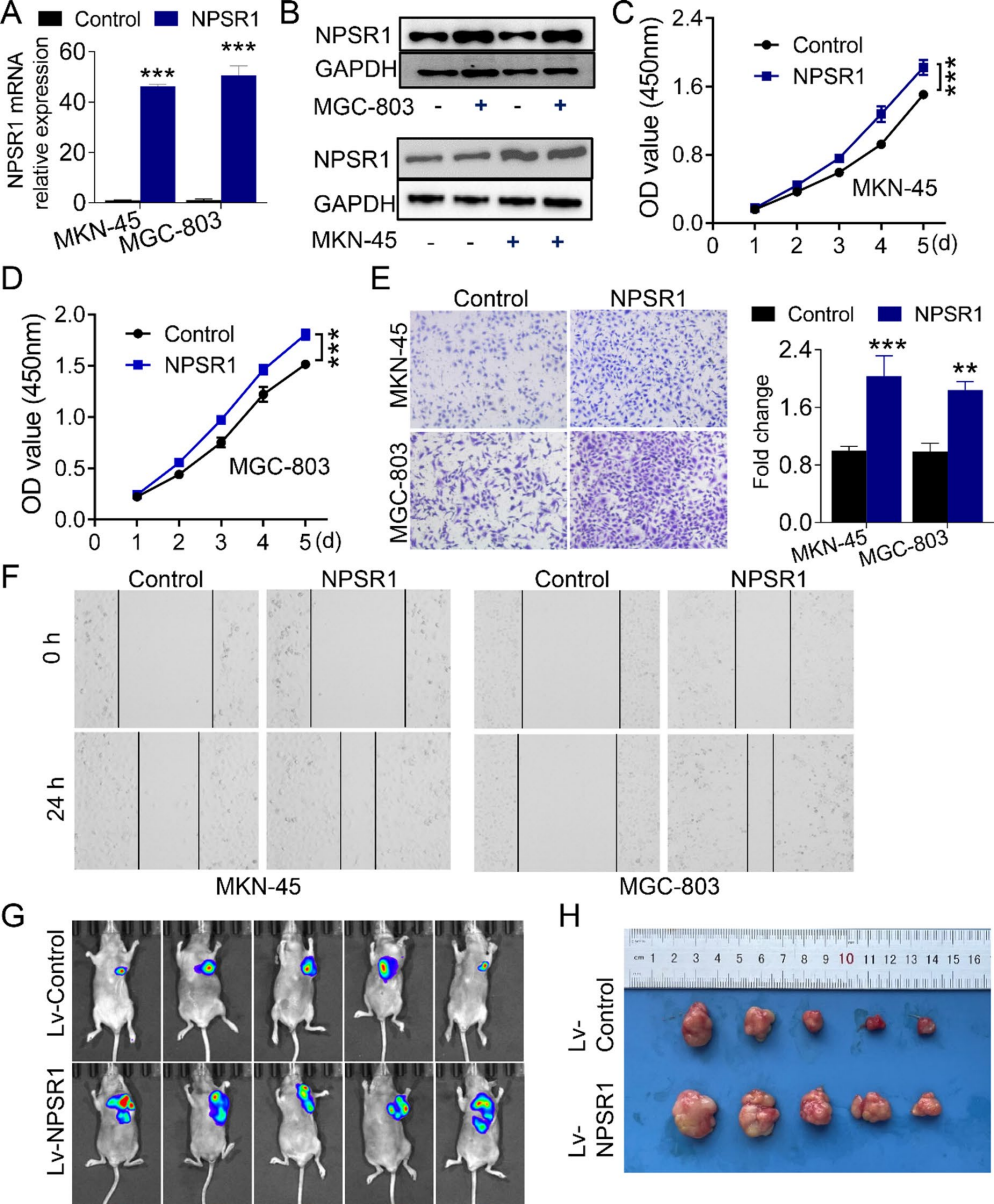
NPSR1 is critical for the cell proliferation and migration of GC. **A**. MKN-45 and MGC-803 cells were transfected with NPSR1, and the efficiency was evaluated by RT-PCR. Student’s t test, ***P < 0.001. **B**. Immunoblots of NPSR1 protein levels in NPSR1-overexpressed MKN-45 and MGC-803 cells. GAPDH was served as a loading control.** C-D**. Cell proliferation was measured by CCK 8 assay from days 1 to 5 in MKN-45 (**C**) and MGC-803 (**D**) cells transfected with NPSR1 plasmid or its control plasmid. Two-way ANOVA, post hoc, Bonferroni, ***P< 0.001. **E-F**. Cell migration ability was assessed in MKN-45 and MGC-803 cells transfected with NPSR1 or its control plasmid by transwell (**E**) and wound-healing assays (**F**), Student’s t test, **P< 0.01, ***P < 0.001. G-H. Tumor volume images were displayed in the LV- NPSR1 group and LV- NPSR1 group from mouse xenograft models at the fifth week

To further demonstrate the oncogenic role of NPSR1 in GC, we performed gain-of-function studies both in vivo and in vitro. First, GC cells were transfected with a plasmid overexpressing NPSR1. Successful transfection was confirmed by RT-PCR and WB analysis (Fig. 2A-B). Then the data from CCK-8 assay revealed that forced NPSR1 expression significantly enhanced the proliferative capacity of MKN-45 and MGC-803 cells (Fig. 2C-D). Moreover, transwell migration and wound healing assays further demonstrated that NPSR1 overexpression significantly increased the migratory capacity of cells (*P* < 0.05), with a marked elevation in migrating cell numbers observed in the NPSR1 overexpressed group compared to controls (Fig. 2E-F). To additionally validate these results, we established a subcutaneous xenograft tumor model to investigate the effect of NPSR1 on tumor growth. GC cells infected with lentiviral vectors encoding either control (Lv-Control) or NPSR1 (Lv-NPSR1) were inoculated into nude mice. The tumor tissues dissected from the nude mice after 5 weeks of implantation, and the tumor volume of Lv-NPSR1 group were significantly bigger compared to the negative control group (Fig. 2G-H). Taken together, the expression of NPSR1 not only facilitates cancer cell proliferation and migration in vitro but also enhances GC growth in vivo.

### NPSR1 silencing inhibits GC cell proliferation and migration

**Fig. 3:**
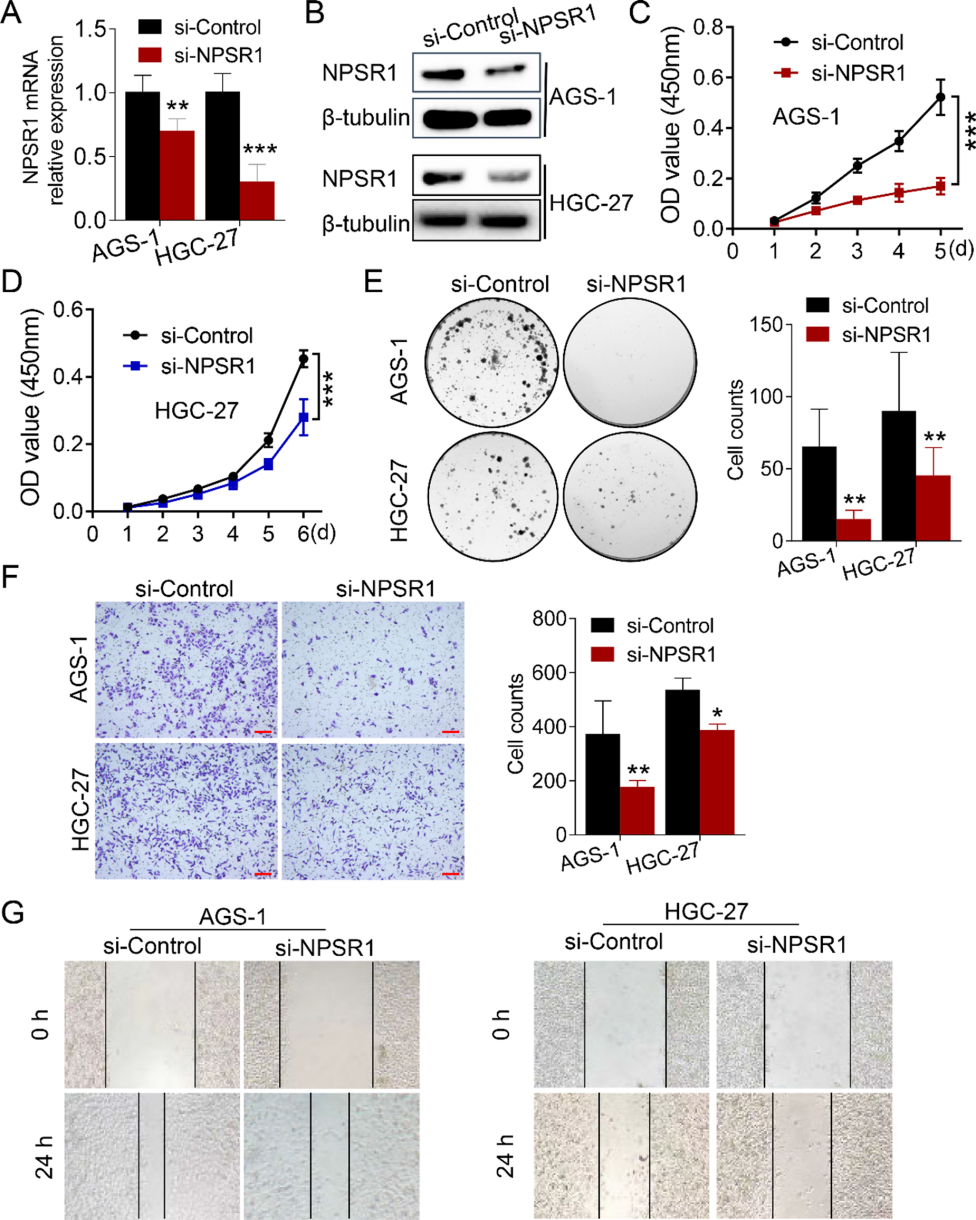
Silencing NPSR1 inhibits the proliferation and migration of GC. **A**. AGS-1 and HGC-27 cells were transfected with NPSR1 siRNA, and the efficiency was evaluated by RT-PCR. **B**. The silencing efficiency was revealed by WB in AGS-1 and HGC-27 cells infected with NPSR1 siRNA or si-Control. **C-E**. Cell proliferation was measured by CCK 8 (**C-D**) and colony formation (**E**) assay in AGS-1 and transfected with NPSR1 siRNA or si-Control. Two-way ANOVA, post hoc, Bonferroni and Student’s t test, **P< 0.01, ***P< 0.001. **F-G**. Cell migration ability was assessed in AGS-1 and HGC-27 cells transfected with NPSR1 siRNA or si-Control by transwell (**F**) and wound-healing assays (**G**), Student’s t test, **P< 0.01, ***P < 0.001. Scale bar: 500 µm

In order to investigate the hypothesis that disturbing NPSR1 could effectively suppress the progression of GC, a siRNA was screened for that could interfere with NPSR1 expression [25]. The effectiveness of the siRNA was confirmed by RT-PCR and WB analysis in AGS-1 and HGC-27 cells (Fig. 3A-B). The results of the CCK8 and colony formation experiments demonstrated that the silencing of NPSR1 significantly reduced the proliferation capacity of the GC cells (Fig. 3C-E). Subsequent analysis was conducted to investigate the impact of NPSR1 knockdown on the migratory behaviour of GC cells. Transwell and wound healing experiments uncovered that the migration ability of GC cells was remarkably reduced upon interference of NPSR1 (Fig. 3F-G). These results suggest that NPSR1 is a viable target for hindering GC progression.

### NPSR1 exerts oncogenic role though dysregulating Hippo pathway

**Fig. 4:**
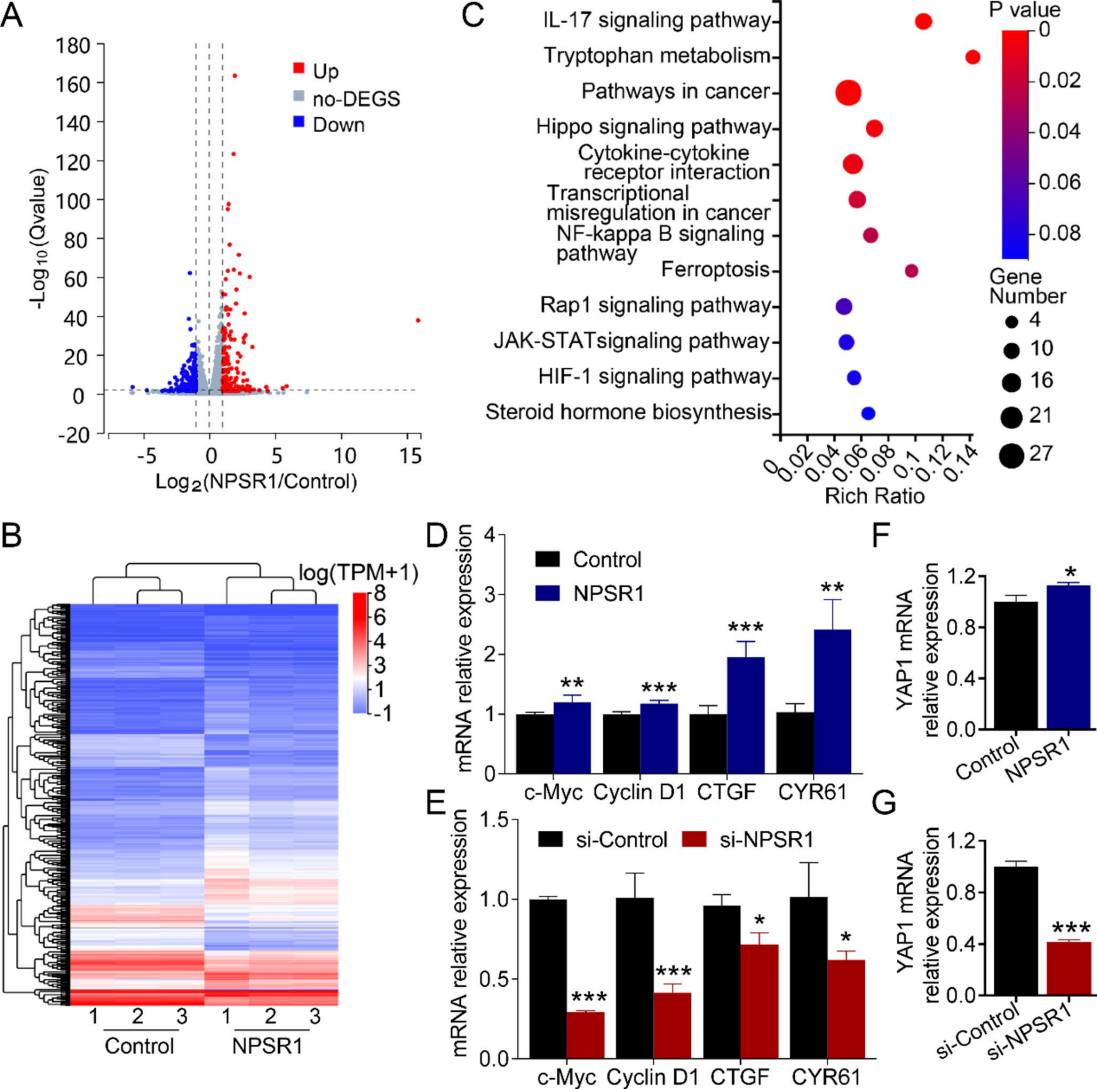
NPSR1 exerts oncogenic role though dysregulating Hippo pathway. **A**. Volcano plot showed the fold change and significance of mRNAs from NPSR1-related differentially expressed genes via RNA sequencing analysis. **B**. Heatmap showed differentially expressed mRNAs after NPSR1 overexpression in MGC-803 cells. **C**. KEGG signaling enrichment analysis showed the downstream pathways mainly regulated by NPSR1. **D**. The mRNA levels of c-Myc, cyclin D1, CTGF and CYR61were examined by RT-PCR after transfected with NPSR1 plasmid or control plasmid in HGC-27 cells, Student’s t test, *P< 0.05, ***P< 0.001. **E**. The mRNA levels of c-Myc, cyclin D1, CTGF and CYR61were examined by RT-PCR after transfected with NPSR1 siRNA or its negative control. Student’s t test, *P< 0.05, ***P< 0.001. **F**. The mRNA level of YAP1 was examined by RT-PCR after transfection with NPSR1 plasmid or control plasmid in HGC-27 cells, Student’s t test, *P< 0.05. **G**. The mRNA level of YAP1 was examined by RT-PCR after transfection with NPSR1 siRNA or its negative control in HGC-27 cells, Student’s t test, ***P< 0.001

The above results indicated that NPSR1 played a significant role in GC progression, and we further exploration was conducted into the molecular mechanisms regulated by NPSR1. In order to address this question, a high-throughput sequencing experiment was initially conducted following the overexpression of NPSR1 in GC cells. The results demonstrated that NPSR1 exerts a significant influence on the expression of numerous genes. We filtered out differentially expressed genes (DEGs) in NPSR1 overexpression group in contrast to the control group, with the criteria for differential expression being a |log2(FC) |>1 and an adjusted p-value < 0.05 (Fig. 4A). Furthermore, bioinformatic analysis indicated that 173 genes were significantly up-regulated, while 264 genes were significantly down-regulated (Fig. 4B). Next, the downstream pathways were analysed by KEGG enrichment, which revealed that Hippo signalling was one of the key downstream pathways of NPSR1 (Fig. 4C; Table 4). Additionally, we examined the genes previously involved in Hippo/YAP1 signaling–mediated GC progression [26, 27] (Table 4). We further validated the expression of these genes enriched in the Hippo signalling pathway in NPSR1-overexpressing and NPSR1-knockdown cells. RT-PCR results indicated that NPSR1 bidirectionally regulated the Hippo signaling pathway (Fig. 4D-E, 8A-B). The hypothesis was formulated that the upregulation of NPSR1 could inactivate Hippo signaling as a key pathway for the transmission of extracellular signals to the cytoplasm, which in turn would trigger the expression of downstream oncogenes in the nucleus. It is evident that YAP1 is the fundamental constituent of the Hippo pathway. To test this hypothesis, the expression of NPSR1 was bidirectionally regulated to ascertain the effect on YAP1. The data from RT-PCR analysis indicated that the mRNA level of YAP1 was increased after NPSR1 overexpression in GC cells (Fig. 4F). ln contrast, knockout of NPSR1 leads to decreased YAP1 transcription (Fig. 4G). In conclusion, these results provide important insights by showing that NPSR1 inactivates the Hippo pathway by regulating its core member, YAP1.

**Table 4:** Transcriptome sequencing revealed that after NPSR1 overexpression, genes enriched in the Hippo signaling pathway via KEGG analysis exhibited significant changes

	Gene ID	Gene Symbol	Log2 (OE / NC)	Qvalue (OE / NC)	
KEEG enrichment	1490	CCN2	1.513485	6.98E-05	CTGF[28]
	154,796	AMOT	-1.69901	9.17E-11	
	1741	DLG3	-1.1038	3.02E-21	
	3993	LLGL2	-1.10856	7.10E-12	
	5054	SERPINE1	2.320624	1.92E-62	
	5521	PPP2R2B	-1.1302	0.039575	
	7475	WNT6	-1.2418	1.20E-12	
	7477	WNT7B	1.635606	8.55E-09	
	81,029	WNT5B	-3.54907	0.036294	
	85,409	NKD2	1.115188	2.80E-04	
	896	CCND3	1.094787	9.20E-23	Cyclin D3
Known GC-associated genes	3491	CCN1	1.559437	3.31E-04	CYR61 [28]
	595	CCND1	0.978948	1.42E-19	Cyclin D1[27]

### NPSR1 promotes YAP1 transcription via cAMP‑CREB signaling

**Fig. 5:**
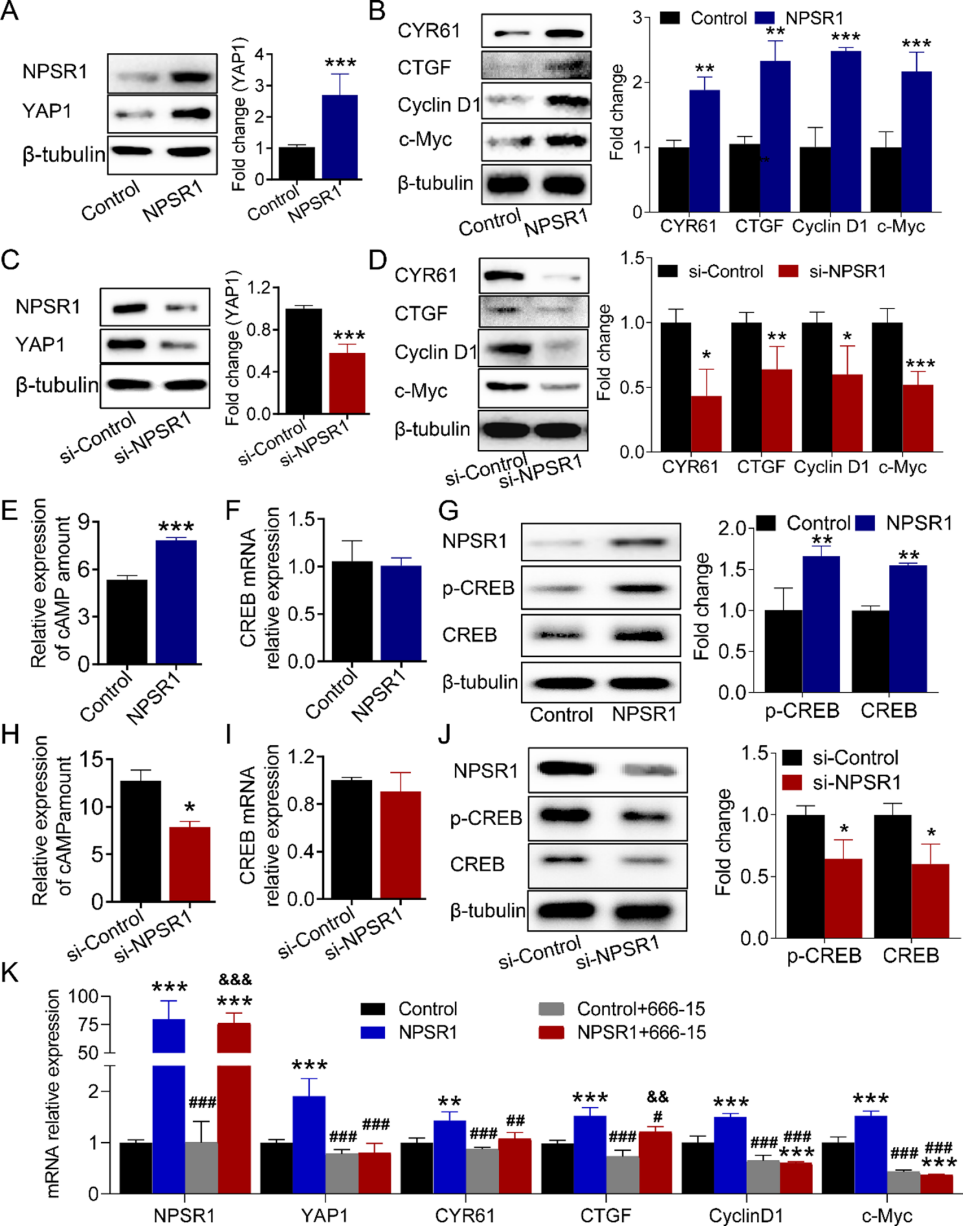
NPSR1 promotes YAP1 transcription by cAMP‑CREB signaling validated in HGC27 cells. **A**. Immunoblots of YAP1 and NPSR1 protein levels after NPSR1 overexpression. **B**. The protein levels of c-Myc, cyclin D1, CTGF and CYR61were examined by WB after transfection with NPSR1 plasmid or control plasmid. Student’s t test, **P< 0.01, ***P< 0.001. **C**. The protein levels of YAP1 and NPSR1 were examined by WB after NPSR1 knockdown. **D**. The protein levels of c-Myc, cyclin D1, CTGF and CYR61were examined by WB after transfection with NPSR1 siRNA or its negative control. Student’s t test, *P< 0.05, **P< 0.01, ***P< 0.001. **E**. ELISA assay detected cAMP amount after overexpression NPSR1. Student’s t test, ***P < 0.001. **F**. The mRNA levels of CREB was examined by RT-PCR. **G**. Immunoblots of CREB and p-CREB protein levels after NPSR1 overexpressed in HGC-27 cells. Student’s t test, **P< 0.01. **H**. ELISA assay detected cAMP amount after inhibiting NPSR1. Student’s t test, *P< 0.05. **I**. RT-PCR showed CREB relative expression after NPSR1suppression. **J**. WB verified CREB and p-CREB protein levels after NPSR1 knockdown. Student’s t test, *P< 0.05. **K**. The mRNA levels of NPSR1, YAP1, c-Myc, cyclin D1, CTGF and CYR61were examined by RT-PCR after transfected with NPSR1 plasmid or control plasmid with or without 20 nm 666-15 in HGC-27 cells. One-way ANOVA, post hoc, Bonferroni, **p < 0.01, ***p < 0.001, (vs. Control); #p < 0.05, ##p < 0.01, ###p < 0.001, (vs. NPSR1); &&p < 0.01, &&&p < 0.001, (vs. Control+666-15)

Recent findings have indicated that c-Myc, cyclin D1, CTGF and CYR61 are pivotal downstream targets of YAP1 and have a significant effect in promoting the progression of GC [12, 13, 26, 27]. We then aimed to ascertain whether the expression of these genes is subject to regulation by NPSR1. Similar to the RT-PCR results (Fig. 4D-G), the WB results indicated that the over-expression of NPSR1 led to a simultaneous increase in the expression of YAP1and its target genes, c-Myc, cyclin D1, CTGF and CYR61, while the knockdown of NPSR1 led in turn to a weakening of their expression (Fig. 5A-D). Moreover, the expression levels of CTGF, c-Myc and cyclin D1 exhibited a notable positive correlation with NPSR1 in human GC tissues (Figure S3A-C). The results provided above demonstrated that NPSR1 is a key upstream regulator of YAP1. We further elucidated the mechanism by which NPSR1 modulates YAP1 expression. It has previously been reported in the literature that NPSR1 could act through Gαs [28, 29], and it is well known that the classical downstream pathway is cAMP/PKA [30]. GO analysis of 49 molecules with more than 4-fold change in expression in HEK293 cells after NPSR1 activation [31] revealed that these molecules were mainly enriched in response to cAMP response (Figure S3D). In addition, the cAMP-PKA-CREB axis has been found to be involved in a multitude of pathological and physiological processes [32], and the transcription factor CREB has been shown to directly regulate the expression of YAP1 once it has entered the nucleus [33]. Thus, we speculated that NPSR1 regulated YAP1 expression by cAMP‑CREB axis. In order to test this possibility, we took the approach of overexpressing NPSR1 or its specific inhibitor (siRNA) in GC cells. ELISA experiments and WB assays demonstrated that overexpression of NPSR1 could promote an increase in cAMP accumulation and enhance the phosphorylation of CREB (Fig. 5E, G). Conversely, knockdown of NPSR1 would reduce cAMP levels and the phosphorylation of CREB (Fig. 5H, J). Furthermore, the regulation of NPSR1 expression does not impact the mRNA level of CREB (Fig. 5F, I). To further validate whether NPSR1 promotes YAP1 transcription dependent on CREB, we employed the CREB inhibitor 666 − 15. The results demonstrated that the regulation of YAP1 and its downstream target genes c-Myc, cyclin D1, CTGF, and CYR61 by NPSR1 was completely blocked by 666 − 15 (Fig. 5K). Collectively, these results demonstrate that NPSR1 disrupts the Hippo pathway by facilitating the transcription of YAP1 via the cAMP-CREB axis.

### NPSR1 mediates YAP1 phosphorylation via PP2Ac

**Fig. 6:**
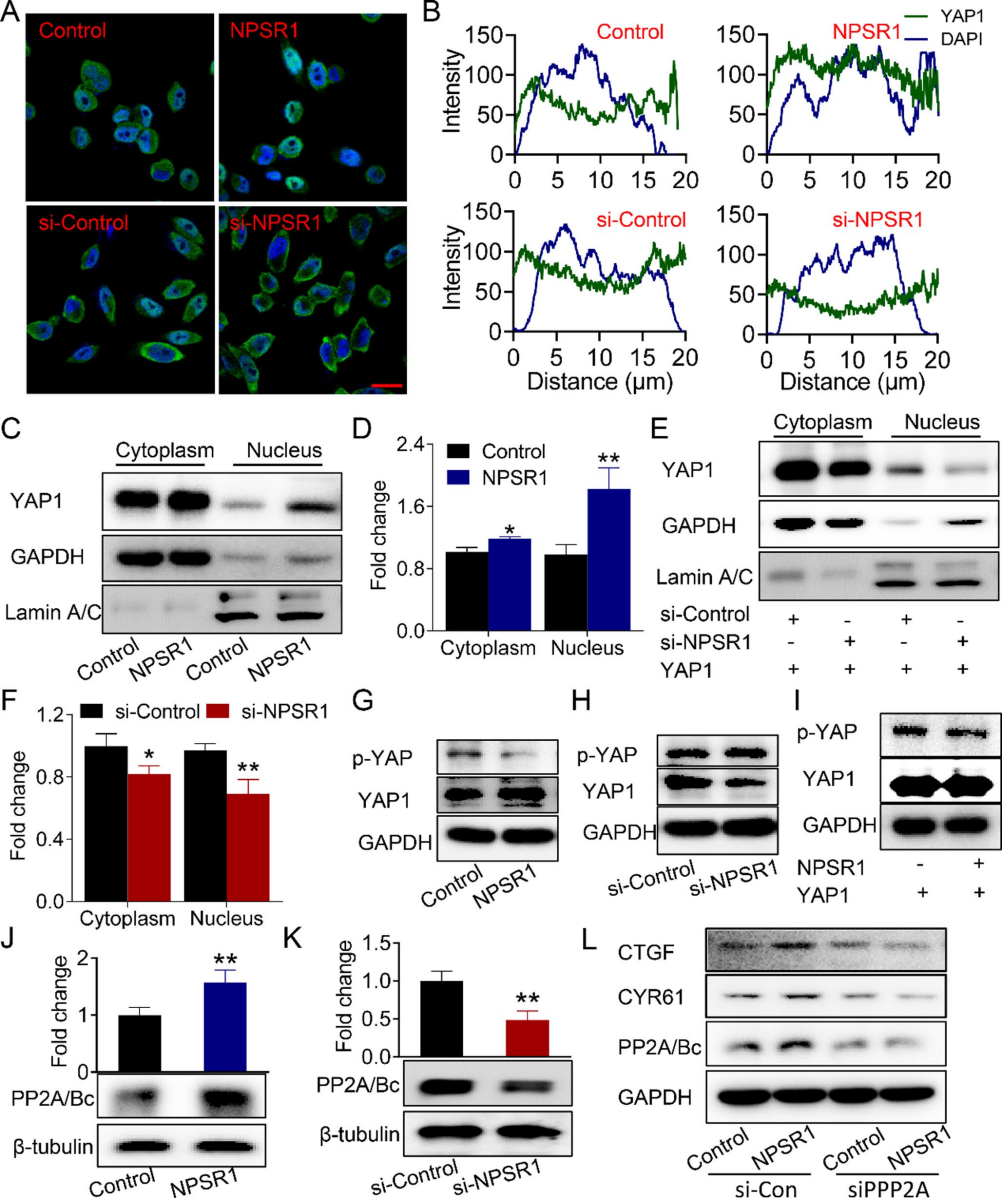
NPSR1 regulates the phosphorylation of YAP1 through PP2Ac. **A**. Immunoblots of YAP1 and NPSR1 protein levels after NPSR1 overexpression. **B**. The protein levels of c-Myc, cyclin D1, CTGF and CYR61were examined by WB after transfection with NPSR1 plasmid or control plasmid. Student’s t test, **P< 0.01, ***P< 0.001. **C**. The protein levels of YAP1 and NPSR1 were examined by WB after NPSR1 knockdown. **D**. The protein levels of c-Myc, cyclin D1, CTGF and CYR61were examined by WB after transfection with NPSR1 siRNA or its negative control. Student’s t test, *P< 0.05, **P< 0.01, ***P< 0.001. **E**. ELISA assay detected cAMP amount after overexpression NPSR1. Student’s t test, ***P < 0.001. **F**. The mRNA levels of CREB was examined by RT-PCR. **G**. Immunoblots of CREB and p-CREB protein levels after NPSR1 overexpressed in HGC-27 cells. Student’s t test, **P< 0.01. **H**. ELISA assay detected cAMP amount after inhibiting NPSR1. Student’s t test, *P< 0.05. **I**. RT-PCR showed CREB relative expression after NPSR1suppression. **J**. WB verified CREB and p-CREB protein levels after NPSR1 knockdown. Student’s t test, *P< 0.05. **K**. The mRNA levels of NPSR1, YAP1, c-Myc, cyclin D1, CTGF and CYR61were examined by RT-PCR after transfected with NPSR1 plasmid or control plasmid with or without 20 nm 666-15 in HGC-27 cells. One-way ANOVA, post hoc, Bonferroni, **p < 0.01, ***p < 0.001, (vs. Control); #p < 0.05, ##p < 0.01, ###p < 0.001, (vs. NPSR1); &&p < 0.01, &&&p < 0.001, (vs. Control+666-15)

The oncogenic activity of YAP1 is predominantly mediated by its nuclear translocation. To explore the role of NPSR1 in modulating YAP1 subcellular distribution, we conducted immunofluorescence and subcellular fractionation assays. Overexpression of NPSR1 significantly elevated both cytoplasmic and nuclear YAP1 levels, with a disproportionately greater increase observed in the nucleus compared to the cytoplasm (Fig. 6A-D). Conversely, NPSR1 knockdown reduced YAP1 abundance in both compartments (Fig. 6A-B). Notably, when YAP1 was experimentally overexpressed, nuclear depletion of YAP1 after NPSR1 suppression showed a more pronounced reduction than its cytosolic counterpart (Fig. 6E-F). We speculated whether NPSR1 regulates YAP1 phosphorylation status to facilitate its nuclear entry. Using a cytoplasm-nucleus separation kit, we discovered that the expression of p-YAP1 in the cytoplasm at site 127 is negatively regulated by NPSR1 (Fig. 6G-H). Moreover, a significant reduction in p-YAP1 levels by NPSR1 was also observed when YAP1 was overexpressed (Fig. 6I). Existing evidence confirms that PP2Ac directly binds YAP1 to dephosphorylate at Ser127 residues [34, 35], a critical step for its nuclear translocation [34]. We next investigated whether NPSR1 mediates PP2Ac expression. Western blot results confirmed that NPSR1 exerts bidirectional regulatory effects on both PP2Ac and PP2Bc expression (Fig. 6J-K). Intriguingly, elevated PP2Ac levels were observed in GC tissues compared to adjacent non-neoplastic samples, and high PP2Ac expression correlated with poor patient prognosis (Figure S4A-B). Importantly, our data uncovered a significant positive correlation between NPSR1 expression and that of PP2Ac and PP2Bc in GC specimens (Figure S4C-D). In order to ascertain whether the phosphorylation of YAP1 by NPSR1 is dependent on PP2Ac, we conducted further experiments using siRNA of PP2Ac in NPSR1-overexpressing cells. These experiments demonstrated that the knockdown of PP2Ac reversed the regulatory effect of NPSR1 on the key downstream target genes of YAP1, namely CTGF and CYR61 (Fig. 6L). Thus, these results demonstrate that NPSR1 promotes the expression of the phosphatase PP2Ac, which binds to and dephosphorylates YAP1, thereby enhancing its nuclear accumulation and promoting the expression of its target genes.

### NPSR1 oncogenic activity relies on YAP1 signaling in GC progression

**Fig. 7:**
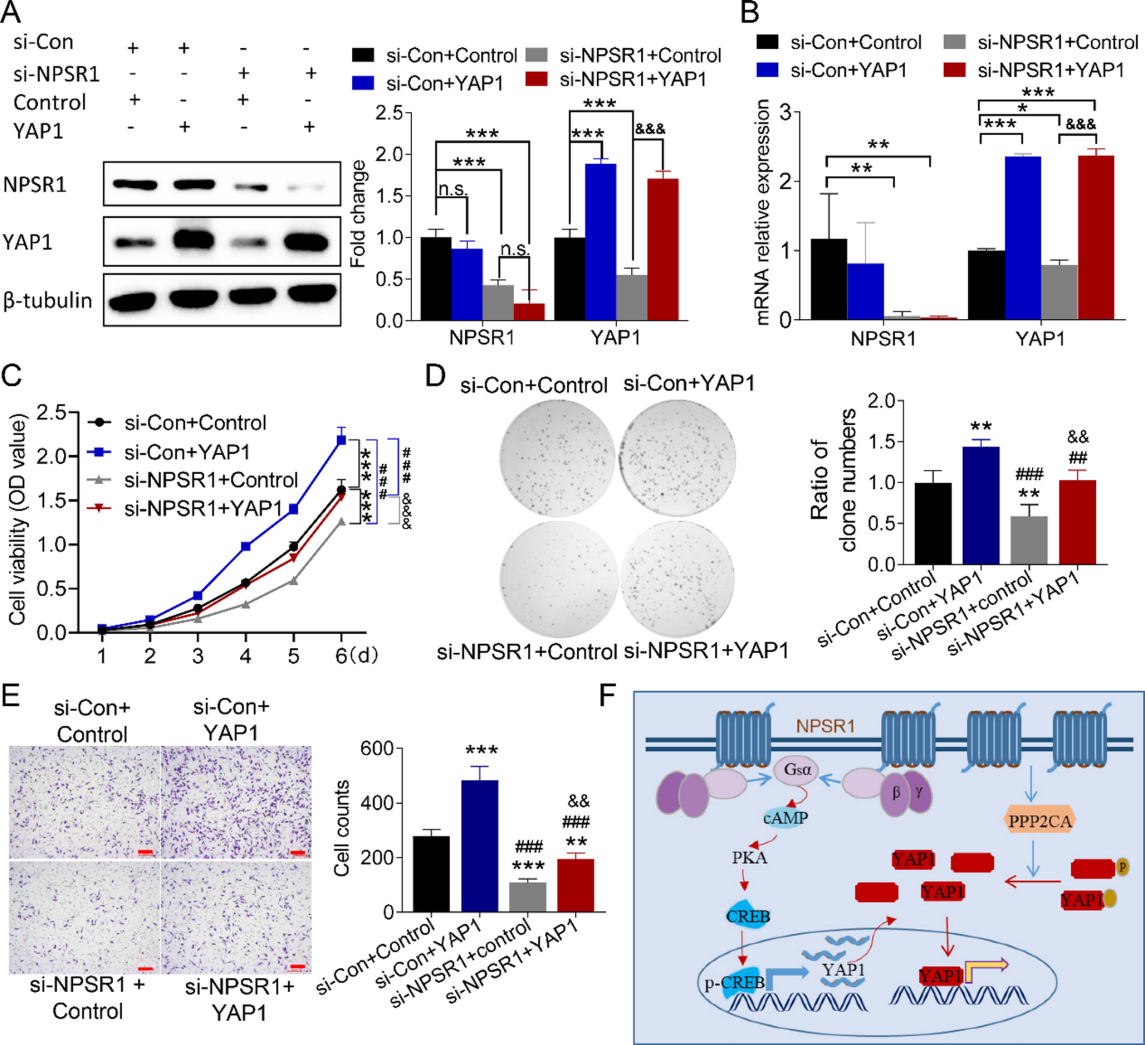
Disturbing YAP1 impairs oncogenic role of NPSR1 in GC. A-B. Protein (**A**) and mRNA (**B**) levels of NPSR1 and YAP1 in HGC-27 cells were exhibited by WB assays and RT-PCR in group of si-Con+ Control, si-Con+ YAP1, si-NPSR1+ Control, or si-NPSR1+ YAP1. β-tubulin served as a loading control. One-way ANOVA, post hoc, Bonferroni, *P< 0.05, **P< 0.01, ***P< 0.001, (vs. si-Con+ Control); &&&P< 0.001, (vs. si-NPSR1+ Control). **C-D**. Cell proliferation was measured by CCK 8 (**C**) and colony formation (**D**) assay in HGC-27 cells that were transfection with si-Con+ Control, si-Con+ YAP1, si-NPSR1+ Control, or si-NPSR1+ YAP1 respectively. One-way ANOVA, post hoc, Bonferroni, **P< 0.01, ***P< 0.001, (vs. si-Con+ Control); ##P< 0.001, ###P< 0.001, (vs. si-Con + YAP1); &&P< 0.01, &&&P< 0.001, (vs. si-NPSR1+ Control). **E**. Cell migration ability was assessed in HGC-27 cells by transwell after transfection with si-Con+ Control, si-Con+ YAP1, si-NPSR1+ Control, or si-NPSR1+ YAP1 respectively. One-way ANOVA, post hoc, Bonferroni, **P< 0.01, ***P< 0.001, (vs. si-Con+ Control); ###P< 0.001, (vs. si-Con + YAP1); &&P< 0.01, (vs. si-NPSR1+ Control). **F**. Model for NPSR1 inactivated the Hippo pathway by enhancing YAP1 transcription and suppressing its phosphorylation

To establish further the relationship between NPSR1 function in GC and the presence of YAP1, rescue experiments were conducted. The primary approach employed was the use of siRNAs to knockdown NPSR1, with and without the concomitant over-expression of YAP1 in GC cells. The data obtained from RT-PCR and WB analysis demonstrated that the silencing of NPSR1 resulted in a reduction in its own expression without affecting the expression of YAP1 in the rescue group of GC cells (Fig. 7A-B). We then observed the proliferation of GC cells, and the results of CCK8 and colony formation assay demonstrated that the multiplication of GC cells was substantially weakened after YAP1 silencing, while such weakening influence was utterly counteracted by the co-transfection of YAP1 (Fig. 7C-D). Moreover, transwell assay outcomes demonstrated that the reduced number of migrated cells caused by NPSR1 knockdown could be completely reversed by increasing expression of YAP1 (Fig. 7E). In summary, the results demonstrated that YAP1 functioned as a key downstream member, facilitating NPSR1 in executing its oncogenic function in GC (Fig. 7F).

## Discussion

The targeting of GPCRs and their associated signaling pathways has emerged as a promising therapeutic strategy for cancer drug development, offering significant potential to improve patient outcomes and enhance the efficacy of existing treatment regimens. Hippo signaling inactivation has been identified as a critical molecular mechanism driving GC progression. However, the precise interaction between GPCRs and the Hippo pathway in promoting GC progression remains poorly understood and warrants further investigation. In this study, we identified G protein-coupled receptor NPSR1 as a key regulator that inactivates Hippo signaling to promote GC progression, marking the first report of NPSR1’s functional role and its underlying mechanism in GC. Our findings reveal a dual regulatory mechanism: first, NPSR1 activates the classical cAMP/CREB signaling axis, thereby enhancing YAP1 transcription; second, NPSR1 promotes the expression of phosphatase PP2Ac, which binds to and dephosphorylates YAP1, thereby enhancing YAP1 activity. (Fig. 7F). These findings not only underscore the critical interplay between GPCRs and the Hippo signaling pathway in GC progression but also establish NPSR1 as a novel oncogene in GC, providing new insights into potential therapeutic targets for GC treatment.

 NPSR1, also designated as GPR154, GPRA (G protein-coupled receptor for asthma) [36], and VRR1 (vasopressin receptor-related receptor 1) [29], represents a multifaceted receptor with diverse physiological functions. Since the identification of its endogenous ligand, neuropeptide S (NPS), in 2004 [28], the NPS/NPSR1 signaling system has been implicated in the regulation of various physiological processes, including arousal, anxiety modulation, learning and memory consolidation, and immunoregulation [22, 37]. Notably, NPSR1 and its ligand exhibit widespread expression across multiple anatomical sites, encompassing both (brain) and peripheral tissues (stomach, thyroid, salivary glands, and mammary glands) [28]. Analysis of RNA expression data from cancer cell lines (https://www.proteinatlas.org/) revealed that NPSR1 expression is particularly prominent in GC cells, ranking highest among 28 cancer cell groups. And elevating NPSR1 levels were observed in pancreatic cancer, bile duct cancer, and colorectal cancer. In this study, we demonstrated that NPSR1 expression is significantly upregulated in GC tissues. While NPSR1 is also highly expressed in various cancerous tissues and cells, further comprehensive investigations are warranted to determine its oncogenic and elucidate the precise molecular mechanisms underlying its role in cancer progression.

GPCRs are integral membrane proteins that facilitate the transmission of extracellular signals to the intracellular compartment. Our microarray analysis showed Hippo signaling was one of the key downstream effects of NPSR1. Conversely, the Hippo signaling transmits cytoplasmic signals to the nucleus, where it exerts a profound influence on the transcription of numerous oncogenes in GC [16]. It was therefore postulated that the observed elevation in NPSR1 expression may result in the inactivation of the Hippo signaling pathway, thereby facilitating the transmission of extracellular signals to the cytoplasm and the subsequent expression of downstream oncogenes in the nucleus. To explore this hypothesis, we examined this in the context of existing literature reports and further analysed high-throughput results with NPSR1 overexpression. The enforced expression of NPSR1 has been demonstrated to serve as a model for ligand-receptor interactions [38]. Therefore, the present study employed NPSR1 overexpression to investigate its function and mechanism. Our results showed that NPSR1 fostered YAP1 transcription by cAMP‑CREB axis in GC cells. This finding is consistent with the results of previous studies that activated NPSR1 increased cAMP production in HEK293 cells [39]. cAMP-PKA-CREB signalling plays a vital role in the process of tumorigenesis and the phosphorylation of Ser133 on CREB is critical for its transcriptional activity [32]. Our data showed that NPSR1 significantly increased the phosphorylation of CREB, which in turn promoted its entry into the nucleus and drove its binding to the YAP1 promoter region to upregulate YAP1 transcription.

In addition, the Hippo pathway includes a kinase cascade that phosphorylates YAP1, thereby inhibiting its translocation to the nucleus. Therefore, exploring whether NPSR1 downstream signalling could disrupt YAP1 phosphorylation is also important. Our data showed that the levels of PP2Ac and PP2Bc were positively regulated by NPSR1. PP2Ac and PP2Bc belong to the C subunit of PP2A [40]. In conjunction with the B and A subunits, they form a heterotrimeric PP2A complex. As a serine/threonine phosphatase, PP2A plays a crucial regulatory role in various cellular processes. Depending on the particular cellular context, each heterotrimeric PP2A holoenzyme exhibits a distinct regulatory profile. PP2A isoforms play a multifaceted role in cancer, functioning as either tumour suppressors or promoters. The present study provides evidence suggesting that PP2Ac acts as an oncogene in the advancement of gastric cancer (GC). Our results showed that NPSR1 decreased the phosphorylation process of YAP1 by increasing PP2Ac, which binds to YAP1 directly and dephosphorylates the serine 127 residue. Further exploration is necessary in order to gain a more comprehensive understanding of the mechanisms through which NPSR1 regulates PP2Ac.

Additionally, our microarray data indicated that NPSR1 regulates several downstream signalling pathways, including the IL-17 pathway and tryptophan metabolism. It is worth further investigating whether NPSR1 mediates these pathways to influence the gastric cancer tumour microenvironment. Our data revealed that inhibiting YAP1/CREB/PP2AC effectively reverses the oncogenic effects of NPSR1. However, in situ tumourigenesis experiments are required to validate whether the pro-oncogenic effects of NPSR1 can be blocked by YAP1/CREB/PP2AC inhibitors. Furthermore, in vivo studies are required to evaluate the efficacy of these inhibitors when used either alone or in combination.

In brief, our results exhibited that NPSR1 functions as a novel oncogene driving the malignant progression of GC. Mechanistically, NPSR1 works as an upstream governor of the Hippo pathway via enhancing YAP1 transcription while simultaneously repressing its phosphorylation, thereby promoting YAP1 nuclear translocation and oncogenic activity. Furthermore, NPSR1 participates in transmitting signals from the cell membrane to the intracellular compartments, ultimately activating the expression of oncogenes associated with GC progression. Our results highlight the value and new insights of NPSR1 as a potential therapeutic target for GC treatment.

## Electronic Supplementary Material

Below is the link to the electronic supplementary material.

Supplementary Material 1 (DOCX 1.62 MB)

## Data Availability

All the datasets generated and analyzed in the present study are available from the corresponding author on reasonable request.

## Abbreviations

GC: Gastric cancer
GPCRs: G protein-coupled receptors
GPR154: G protein-coupled receptor 154
NPSR1: Neuropeptide S receptor1
NPS: Neuropeptide S
TCGA: The Cancer Genome Atlas
OS: Overall survival
cAMP: Cyclic adenosine monophosphate
CREB: cAMP-responsive element binding protein
PP2A: Protein phosphatase 2 A
PP2Ac: Protein Phosphatase 2 Catalytic Subunit Alpha PPP2CA
