# Supplementary material for: Targeting NPSR1-mediated Hippo-YAP1 dysregulation suppresses gastric cancer progression
Source: Cell Mol Life Sci. 2026 Apr 9;83(1):217. doi: 10.1007/s00018-026-06181-6 (PMC13187076; doi:10.1007/s00018-026-06181-6)
Supplement: Supplementary file 1 — Supplementary Material 1 (DOCX 1.62 MB) [file 18_2026_6181_MOESM1_ESM.docx]

**Targeting NPSR1-Mediated Hippo-YAP1 Dysregulation Suppresses Gastric Cancer Progression**

Wenjing Qin ^1#^, Mei Ma^2#^, Weidan Fang^3^, Xian Wang^3^, Bin Yu^4^

^1^ Department of General Surgery, The First Affiliated Hospital, Jiangxi Medical College, Nanchang University, Nanchang, 330006, PR China. ORCID: 0009-0003-8733-8759.

^2^ Department of Oncology, The First Affiliated Hospital, Jiangxi Medical College, Nanchang University, Nanchang, 330006, PR China. ORCID: 0000-0002-4656-3752.

^3^ Department of Oncology, The First Affiliated Hospital, Jiangxi Medical College, Nanchang University, Nanchang, 330006, PR China.

^4^ Department of General Surgery, The First Affiliated Hospital, Jiangxi Medical College, Nanchang University, Nanchang, 330006, PR China. yubin@ncu.edu.cn. ORCID: 0000-0003-1567-8415.

^#^ These authors contributed equally: Wenjing Qin, Mei Ma

Corresponding author: Bin Yu

**Figures S1-S4**


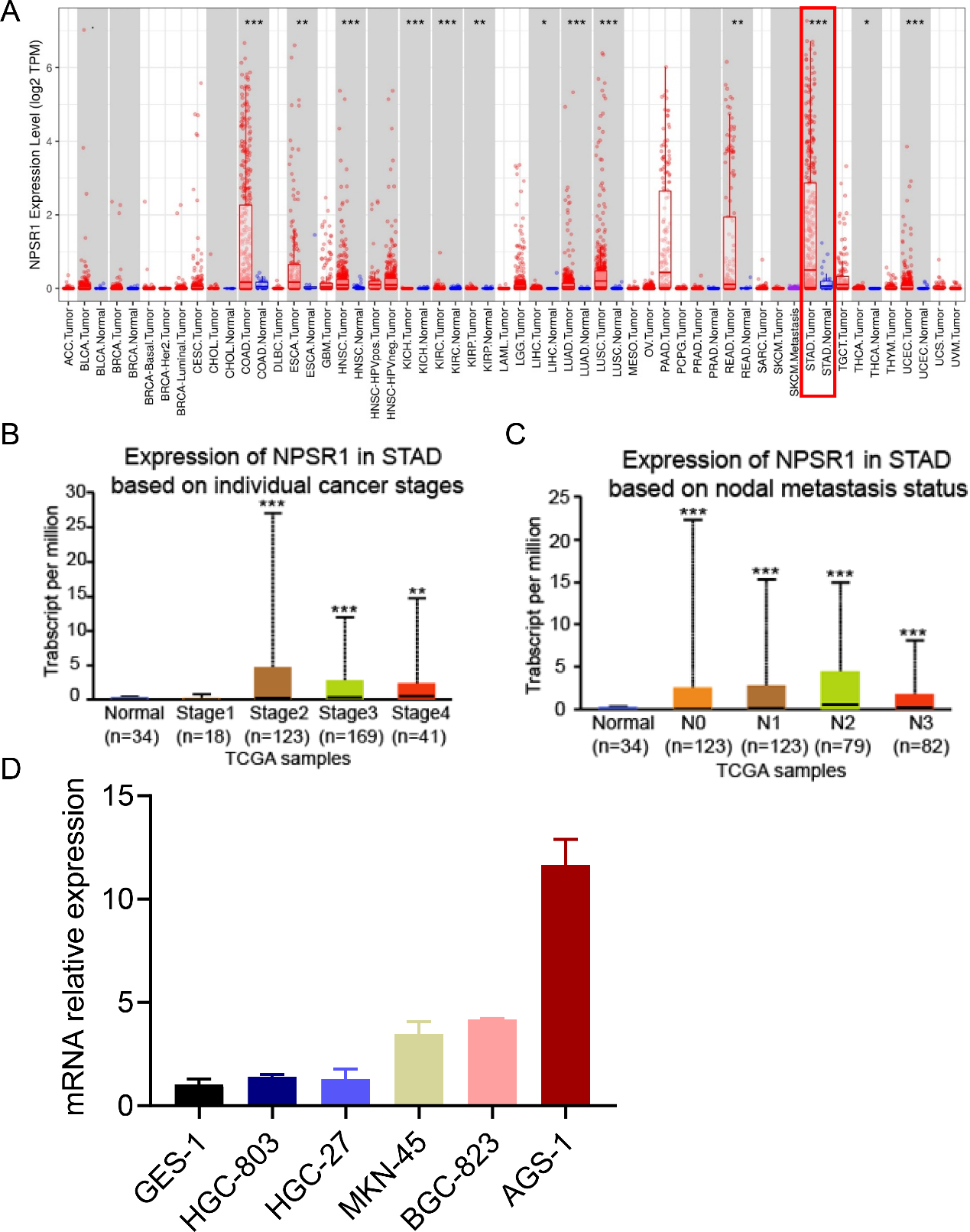


**Figure.S1. Expression of NPSR1 in gastric cancer tissues and cells.**

1. The expression profile of NPSR1 across 55 tumor and adjacent normal tissues was analyzed by the TIMER2.

**B-C.** Association of NPSR1 expression with GC stage (B) and nodal metastasis status (C) in patients. One-way ANOVA, post hoc, Bonferroni, **P< 0.01, ***P< 0.001.

**D.** RT-PCR detected NPSR1 expression levels in GC cells and normal gastric cells.


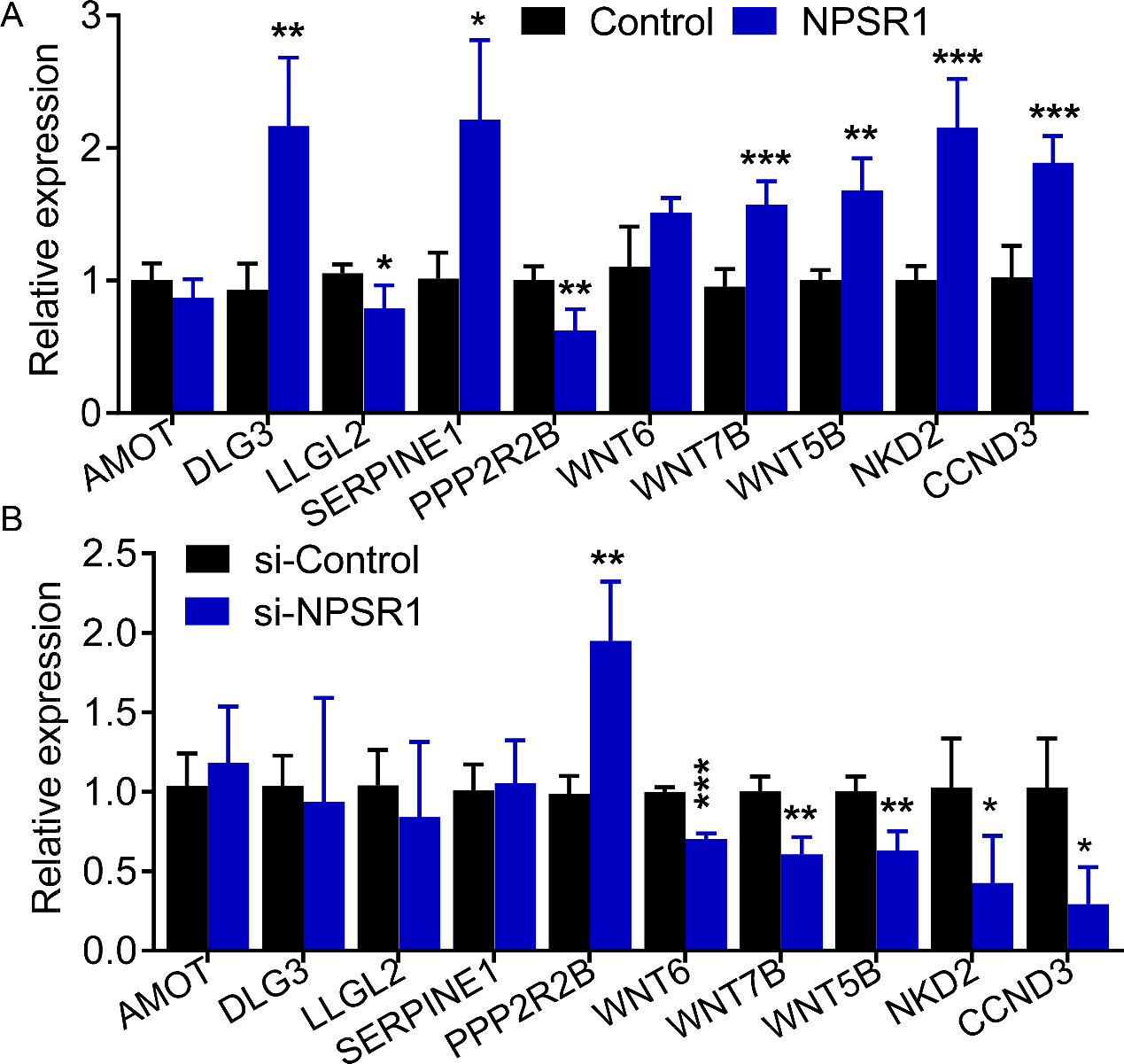


**Figure.S2.** RT-PCR results showed NPSR1 regulated genes expression in the Hippo signaling pathway.

**A.** The mRNA levels of genes enriched in the Hippo signalling pathway were examined by RT-PCR after transfection with NPSR1 plasmid or control plasmid in HGC-27 and MGC-803 cells. Student’s t test, *P< 0.05, **P< 0.01, ***P< 0.001.

**B.** The mRNA levels of genes enriched in the Hippo signalling pathway were examined by RT-PCR after transfection with NPSR1 siRNA or its negative control in HGC-27 and MGC-803 cells. Student’s t test, *P< 0.05, **P< 0.01, ***P< 0.001.


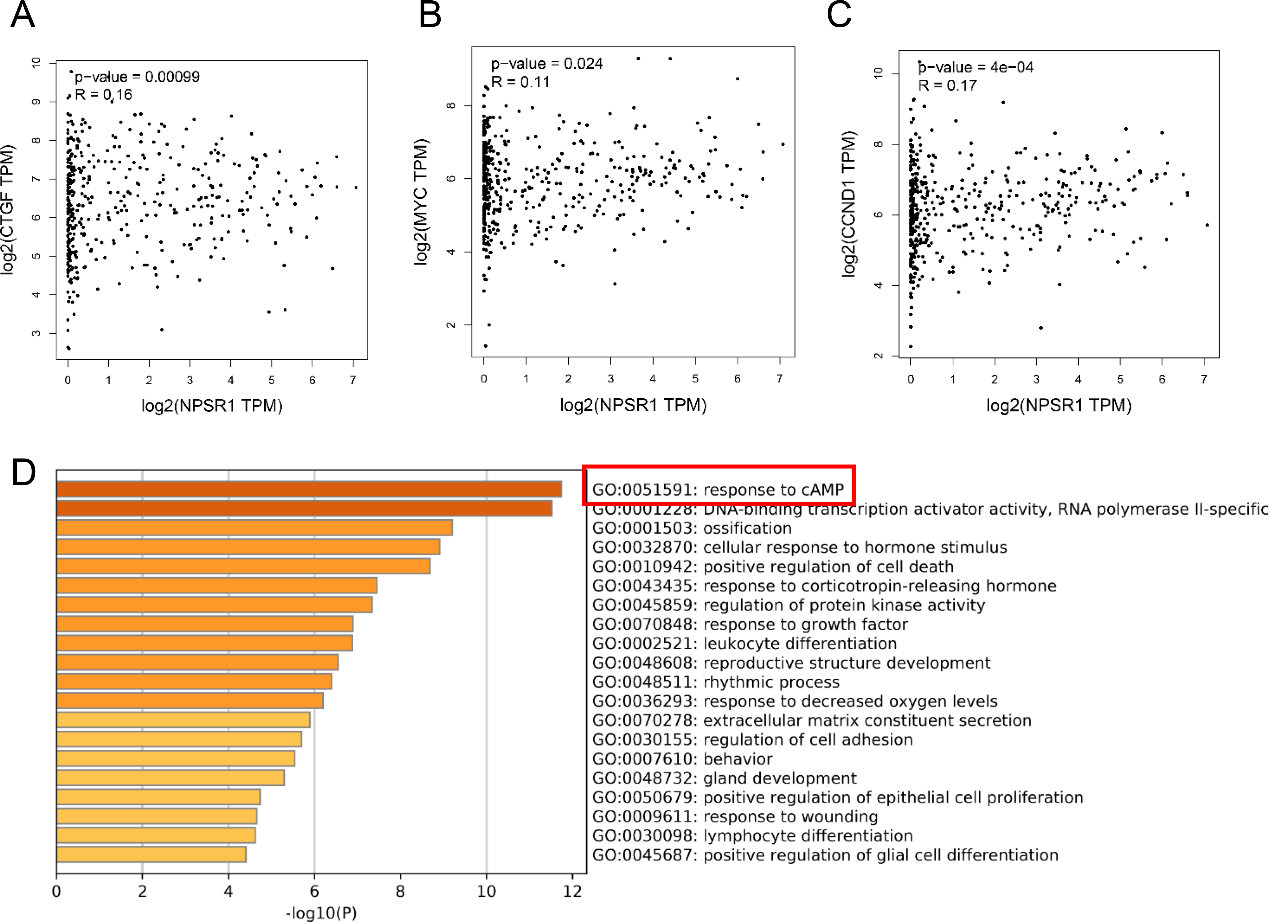


**Figure.S3.** **NPSR1 controls essential downstream pathways and key molecules.**

**A-C.** Correlation analysis indicates that NPSR1 expression in GC is significantly associated with the expression of CTGF, c-Myc, and cyclin D1.

**D.** GO analysis of the major enrichment pathways of NPSR1 downstream target genes.


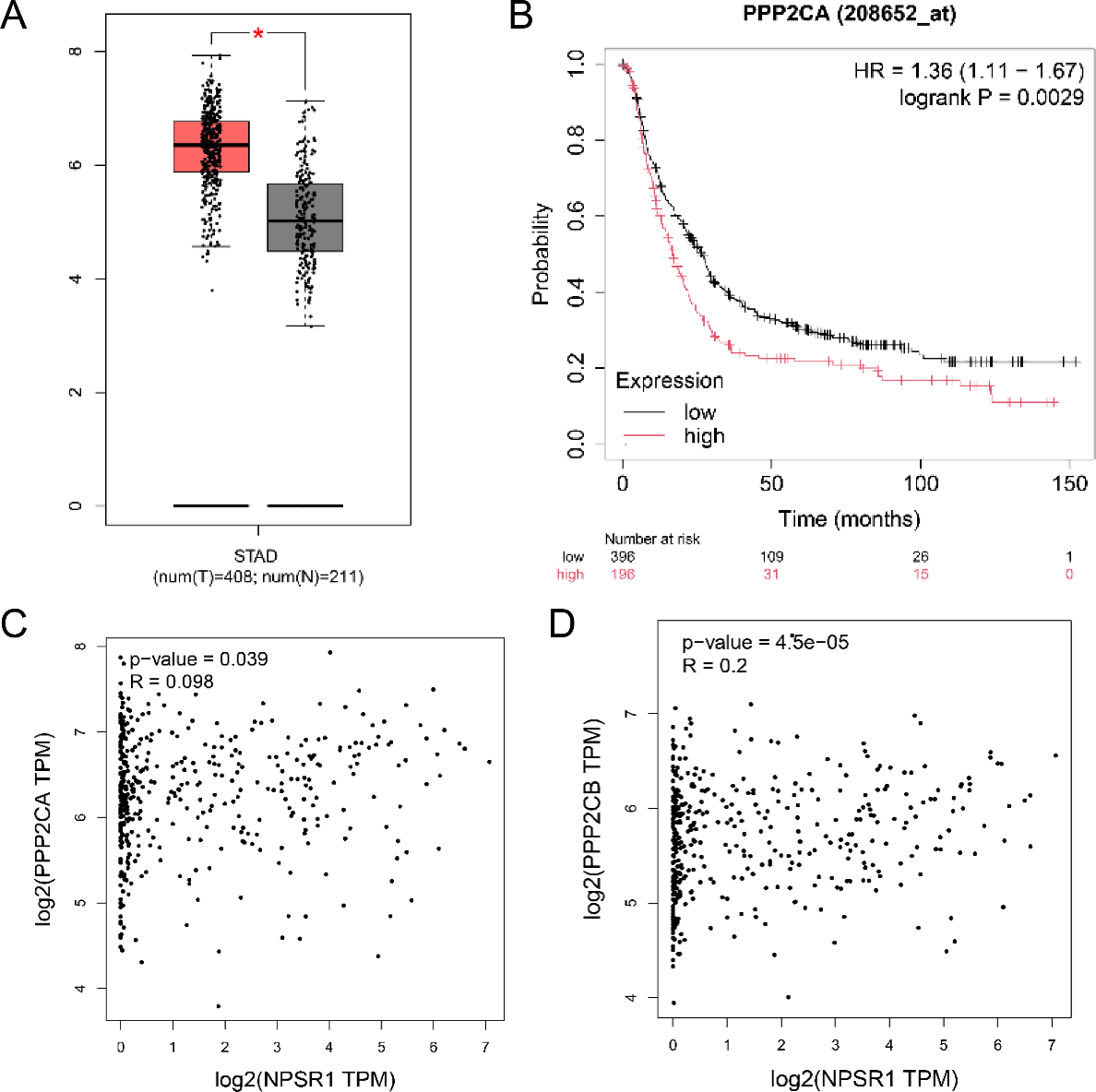


**Figure.S4.** **PP2A expression and its link to prognosis and NPSR1 in GC.**

1. Boxplot exhibited the expression level of PP2Ac in GC tissues compared to its adjacent non-cancerous tissue. Student’s t test, *P < 0.05.
2. The Kaplan–Meier survival analysis was conducted in accordance with PP2Ac expression in GC.

**C-D.** The GEPIA database demonstrates the correlation between NPSR1 expression and the expression of PP2Ac and PP2Bc in GC.

**Tables S1-S2**

**Table S1. Primers used in this study.**

| Genes | Forward/Reverse | 5’ to 3’ |
| --- | --- | --- |
| NPSR1 | For | CATTCTTGCCTTCATCTGCTGTT |
|  | Rev | CTCCTGGGTGTCTGGAAGGA |
| YAP1 | For | CAATAGCTCAGATCCTTTCCT |
|  | Rev | TAGTATCACCTGTATCCATCTC |
| c-Myc | For | AGCCTCCCGCGACGAT |
|  | Rev | GAGTCGTAGTCGAGGTCATAGTTCCT |
| Cyclin D1 | For | CAGAGGCGGAGGAGAACAA |
|  | Rev | ATGGAGGGCGGATTGGAA |
| GAPDH | For | ATCATCCCTGCCTCTACTGG |
|  | Rev | TGGGTGTCGCTGTTGAAGTC |
| CREB1 | For | ATTCACAGGAGTCAGTGGATAGT |
|  | Rev | CACCGTTACAGTGGTGATGG |
| CYR61 | For | CGAGGTGGAGTTGACGAGAA |
|  | Rev | GCACTCAGGGTTGTCATTGGT |
| CCN2 | For | GGGAAATGCTGCGAGGAGT |
|  | Rev | CTTCCAGTCGGTAAGCCGC |
| AMOT | For | CATGGAGGGCAGGATTAAGACC |
|  | Rev | CGACAGCTGCTCTGTCTTGCT |
| DLG3 | For | GCTGGCGGTGAACAAC |
|  | Rev | GGCTGGAATTATGGCTTAT |
| LLGL2 | For | TTTAACAAGACGGTGGAGCA |
|  | Rev | GAGCTTGATGGCTCCAGAAC |
| SERPINE1 | For | AGTGGACTTTTCAGAGGTGGA |
|  | Rev | GCCGTTGAAGTAGAGGGCATT |
| PPP2R2B | For | ATCCTGCCACCATCACAAC |
|  | Rev | GCGTTGGCAAATACTCTTCG |
| WNT6 | For | TCCGCCGCTGGAATTG |
|  | Rev | AGGCCGTCTCCCGAATG |
| WNT7B | For | GTGAAGCTCGGAGCACTG |
|  | Rev | ACTGGTACTGGCACTCGTTG |
| WNT5B | For | GACGGGTGACAGAGGGAAC |
|  | Rev | TGCACCGGGTTCAAAGCTAA |
| NKD2 | For | AGCGCAGATGACGGAGAGAGA |
|  | Rev | CGAGACATCGCACTGGAGT |
| CCND3 | For | GGTGCAATCCTCTCCTCGC |
|  | Rev | TAGTTCATGGCCAGGGGGAA |

**Table S2. Antibodies used in this study**.

| Antigens | Manufacturers | Application |
| --- | --- | --- |
| NPSR1 | Abcam, ab92425 | WB, 1:1000; IF/IHC,1:50 |
| GAPDH | Proteintech, 81640-5-RR | WB, 1:1000 |
| YAP1 | Cell signaling technology, 4912 | WB, 1:1000; IF,1:50 |
| β-tubulin | OriGene, TA503129 | WB, 1:1000 |
| p-CREB | Proteintech, 28792-1-AP | WB, 1:1000 |
| CREB | Proteintech, 12208-1-AP | WB, 1:1000 |
| c-Myc | Cell signaling technology, 9402 | WB, 1:1000 |
| Cyclin D1 | Cell signaling technology, 2922 | WB, 1:1000 |
| PP2A/Bc | Abcam, ab32065 | WB, 1:1000 |
| p-YAP1 (Ser127) | Proteintech, 80694-2-RR | WB, 1:1000 |
| CTGF | Proteintech, 25474-1-AP | WB, 1:1000 |
| CYR61 | Proteintech, 26689-1-AP | WB, 1:1000 |
